# Scedosporium apiospermum: A Rare Cause of Aggressive Orbital Apex Syndrome

**DOI:** 10.7759/cureus.3743

**Published:** 2018-12-17

**Authors:** Ui Lyn Loh, Pih Yih Tai, Adil Hussein, Fazilawati A Qamarruddin

**Affiliations:** 1 Ophthalmology, Universiti Sains Malaysia, Kota Bharu, MYS; 2 Ophthalmology, Hospital Tengku Ampuan Rahimah, Klang, MYS

**Keywords:** scedosporium apiospermum, orbital apex syndrome

## Abstract

Orbital apex syndrome (OAS) is a localized orbital cellulitis at the orbital apex that can cause vision loss from optic neuropathy and ophthalmoplegia involving multiple cranial nerves. Herein, we report a rare and rapidly progressive case of OAS secondary to fungal pansinusitis caused by *Scedosporium apiospermum* in an immunocompromised patient following the extraction of abscessed teeth.

A 48-year-old man with diabetes mellitus who had failed to adhere to his treatment presented with complaints of a right-sided headache and toothache for two weeks, with nausea and vomiting for two days prior to presentation. The patient was treated for septic shock secondary to the dental abscesses. Non-contrast brain computed tomography (CT) showed no significant intracranial abnormalities other than pansinusitis. Four days later, dental extraction was performed. The patient reported progressive painless blurring of the vision in his right eye following the dental extractions and was referred to the ophthalmology department. Subsequent examinations revealed decreased optic nerve function and ophthalmoplegia in his right eye and dental caries in the upper molars, with a mucopurulent discharge from the right sphenoid region. The clinical diagnosis was OAS. Pus near the orbital apex was drained surgically. Methicillin-resistant *Staphylococcus aureus* was isolated from the pus and a nasal swab. Tissue culture from the septal wall yielded *S. apiospermum*. The patient’s condition deteriorated, despite intensive antibiotic and antifungal treatment and repeated surgical debridement. The disease progressed rapidly to his left eye. Sixty-seven days after the inital presentation, his visual acuity (VA) of both eyes was classified as no perception of light (NPL). The patient discharged himself from the hospital (at own risk discharge) and subsequently failed to attend a scheduled appointment in the ophthalmology clinic.

If immunocompromised patients present with OAS, fungal infections should be ruled out. Prompt and aggressive treatment using a multidisciplinary approach is mandatory in cases of potentially life-threatening and vision-threatening fungal infections.

## Introduction

Orbital apex syndrome (OAS) consists of a combination of oculomotor, trochlear and abducens cranial nerve paresis, the ophthalmic branch of the trigeminal cranial nerve distribution sensory loss and vision loss due to optic nerve involvement. *Scedosporium apiospermum*, a filamentary fungus, is a rare cause of ocular infections. Ocular infections caused by *S. apiospermum* pose a therapeutic challenge due to its high resistance to antifungal agents and rapid proliferation, which leads to high morbidity and mortality [1]. In published case series, keratitis, sclerokeratitis and scleritis were reported to be the most common presentations of *S. apiospermum* ocular infection [2-3]. Herein, we describe an uncommon case of an invasive *S. apiospermum* infection causing OAS in an immunocompromised patient.

## Case presentation

**Figure 1:**
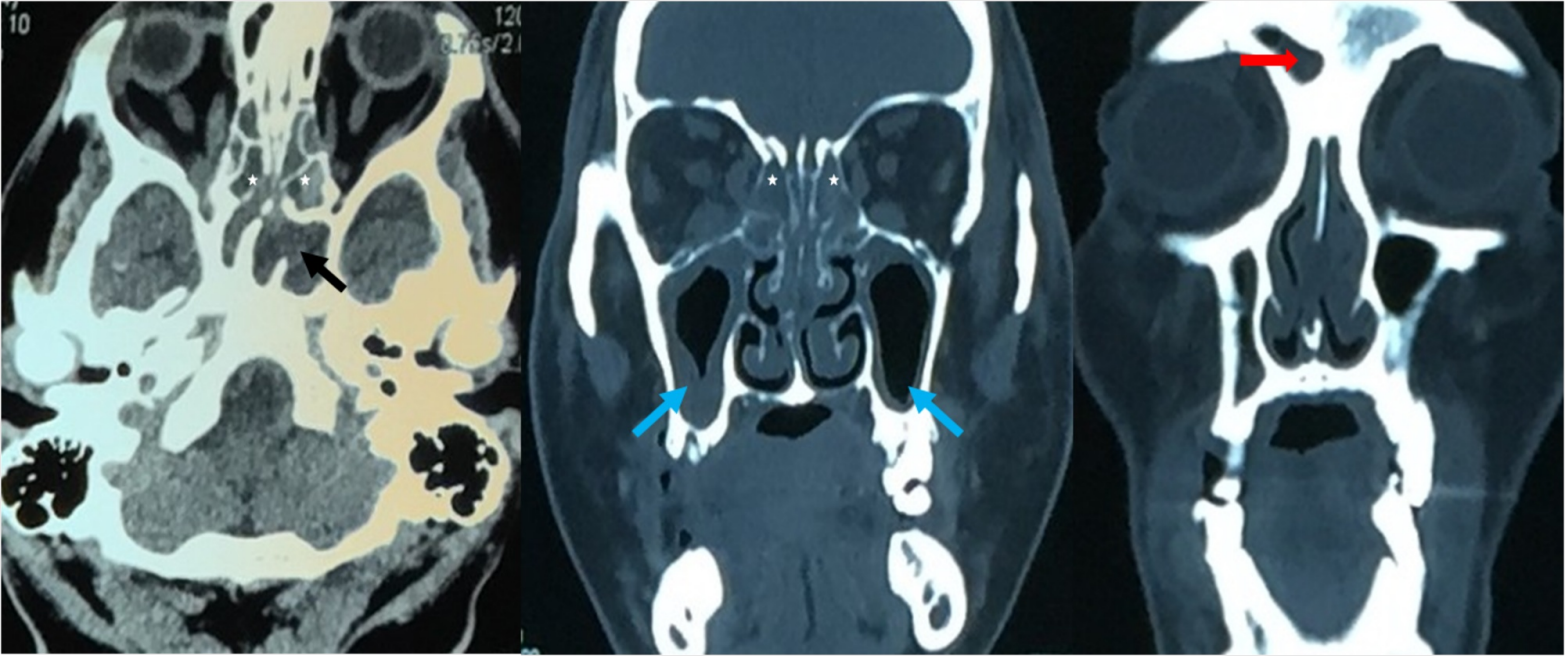
Non-contrasted CT on initial presentation showed mucosal thickening in ethmoid sinus [white star on axial and coronal plane], sphenoid sinus [black arrow on axial plane], maxillary sinus [blue arrow on coronal plane] and frontal sinus [red arrow on coronal plane] indicating pansinusitis CT: computed tomography

A 48-year-old man with diabetes mellitus who had failed to adhere to his treatment presented to the emergency department with complaints of a right-sided headache and toothache for two weeks, with nausea and vomiting for two days prior to presentation. The patient was managed for septic shock secondary to dental abscesses. Non-contrast brain computed tomography (CT) was performed. The CT scan showed no significant intracranial abnormality other than pansinusitis, without obvious retro-orbital fat streakiness (Figure 1). Four days later, tooth number 17 and 18 in the left mandibular region, both of which had abscesses, were extracted. The patient noted progressive painless blurring of the vision in his right eye post-extraction of the abscessed teeth and was referred to the ophthalmology team two days later. At this time, the patient was receiving day five of treatment with intravenous (IV) amoxicillin-clavulanate, (1.2 g three times a day), with the addition of IV metronidazole (400 mg twice a day).

The visual acuity (VA) in his right eye was 4/60, with no improvement in the pinhole test. A relative afferent pupillary defect was present, with an associated decrease in the optic nerve function, in addition to ophthalmoplegia and restrictions in the superior and lateral gaze. No proptosis, ptosis or lid swelling was present, and all other anterior segment findings were normal. A fundus examination of the right eye showed only proliferative diabetic retinopathy. The VA in the patient’s left eye was 6/18 and 6/9 in the pinhole test. An examination of the anterior segment was unremarkable. Moderate non-proliferative diabetic retinopathy was detected in the posterior segment. Dental caries were present in the upper molars, with mucopurulent discharge from the right sphenoid sinus region. The clinical diagnosis was OAS, and emergency functional endoscopic sinus surgery and septoplasty were performed by the ear, nose and throat (ENT) team. Intra-operatively, pus (6cc) was drained from the right sphenoid sinus. The pus breached the anterior sphenoid wall, tracked down to the septum polypoidal mucosa over the right maxillary sinus and right anterior and posterior ethmoid sinuses.

**Figure 2:**
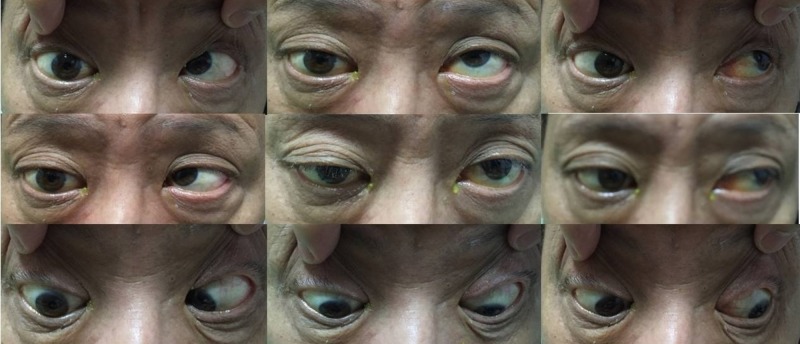
Nine cardinal gazes of patient at day 4 of presentation with restriction of all gazes in the right eye

Empirical treatment with IV ceftriaxone (1 g twice a day) and IV fluconazole (400 mg once a day) was started. Despite the commencement of IV antibiotic and antifungal treatment, the patient’s condition deteriorated, and the VA in this right eye was classified as no perception of light (NPL) and almost total ophthalmoplegia four days post-presentation (Figure 2). Treatment with IV methylprednisolone was not started in view of the infective nature of the disease and the possibility of a fungal infection, which could worsen his condition.

**Figure 3:**
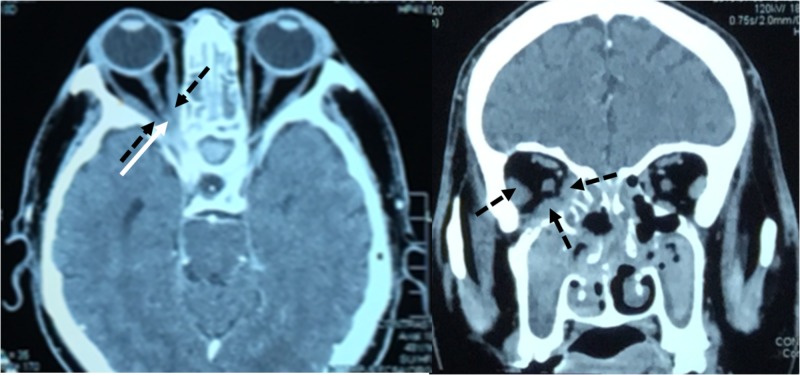
Contrast-enhanced CT of brain and orbit 12 days post initial CT scan showed thickened and enhanced right optic nerve at the right orbital apex [white arrow]. The medial, inferior and lateral rectus muscles appear thickened [black arrow], predominantly at the orbital apex. CT: computed tomography

Methicillin-resistant *Staphylococcus aureus* was isolated from the pus and from a nasal swab. IV vancomycin (1 g twice a day) and IV metronidazole (400 mg twice a day) were added. An urgent non-contrast CT brain scan was repeated and revealed features suggestive of fungal pansinusitis, with the involvement of the right orbital apex and right orbital contents (Figure 3). No suspicious intracranial lesion was seen. The patient underwent emergency nasal debridement for the second time and septotomy. A bony dehiscence was noted over the right posterior ethmoid supralaterally, exposing the orbital apex.

On day 13 after the first surgical drainage, tissue culture from the septal wall yielded *S. apiospermum*. IV amphotericin B (35 mg once a day) was commenced. However, the patient’s renal profile deteriorated following the amphotericin B treatment. Thus, the antifungal regime was changed to oral voriconazole (200 mg twice a day), but the patient failed to respond to the treatment. Forty days after the initial presentation, he developed invasive pansinusitis, with disease progression involving the base of the skull (both orbits) and intracranial extension.

At this stage, the VA in the patient’s right eye was classified as NPL in all four quadrants, with almost total ophthalmoplegia. The VA in his left eye was 6/12, with full extraocular movement. The neurosurgical team decided against any surgical intervention. Functional endoscopic sinus surgery was performed for the third time, with nasal toileting. Intra-operatively, minimal crusting was observed over the right nasal cavity. The right sphenoid, pterygoid, maxillary sinuses and the frontal sinuses were clear, with no pus. The mucosa was healthy, with minimal bleeding. The left maxillary sinus was also clear.

**Figure 4:**
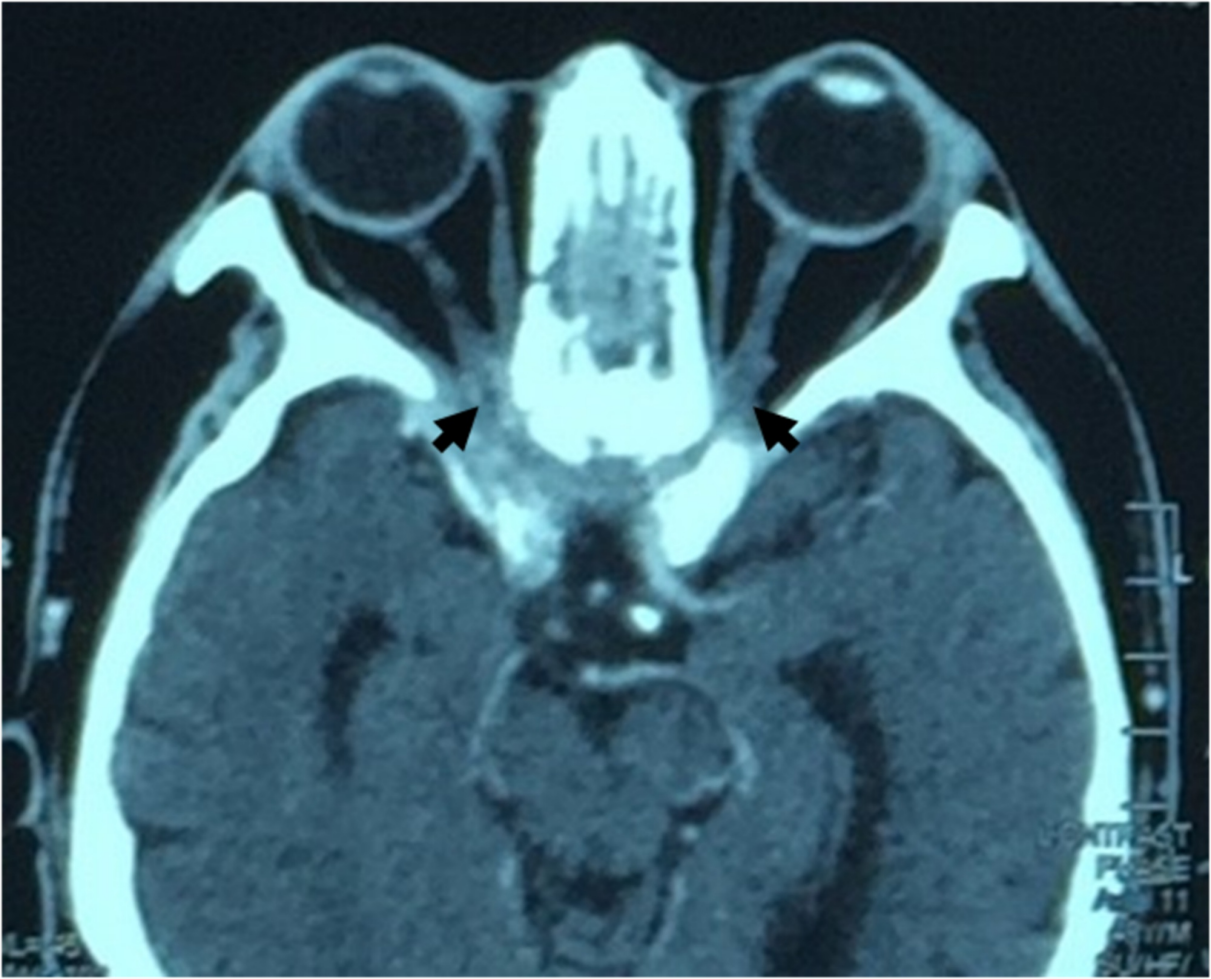
Contrast-enhanced CT of orbit noted extension of enhancement and thickening at bilateral orbital apex [black arrow] CT: computed tomography

IV amphotericin B was restarted, but the patient developed gastrointestinal intolerance. The antifungal treatment was again changed to oral voriconazole (200 mg twice a day). Fifty-seven days after the initial presentation, the VA in the patient’s left eye decreased suddenly to 6/30 (reduced Snellen chart), and his colour vision decreased. A plain and contrast-enhanced CT scan of the brain, orbit and the paranasal sinuses showed invasive pansinusitis, with bilateral orbital apex involvement (Figure 4). It also showed an increasing intracranial extension involving the right cavernous sinus anteriorly, the roof of the sphenoid sinus, the floor of the anterior cranial fossa and right medial cranial fossa anteriorly, as well as osteomyelitis changes at the base of the skull. In view of the acute progression of the infection, surgical intervention was considered. However, the area of the lesion was too extensive and was beyond ophthalmology coverage. Following a reassessment by the neurosurgical team, a decision was made to continue medical treatment rather than perform a surgical intervention.

SIxty-seven days after the initial presentation, the patient’s VA decreased to NPL in both eyes. Although his Glasgow coma scale was full, his general condition worsened, with lethargy, irritability and dizziness, accompanied by nausea and vomiting. The patient decided to discharge himself from the hospital (at own risk discharge) and subsequently failed to attend an appointment in the ophthalmology clinic.

## Discussion

*Scedosporium apiospermum*, the asexual (anamorph) stage of *Pseudallescheria boydii*, is an emerging opportunistic mold infection in immunocompromised and occasionally immunocompetent patients [4]. It is a hyaline filamentous fungus, which is present in the soil, sewage and polluted waters [5]. In normal hosts, it usually produces localized disease after penetrating trauma or aspiration of polluted water [6]. Ocular infections induced by *S. apiospermum* include keratitis, conjunctival mycetoma, endophthalmitis and panophthalmitis. Reports of orbital involvement are rare [7]. In immunocompromised patients, *S. apiospermum* may cause severe pulmonary or resistant life-threatening disseminated infections [6,8].

OAS associated with *S. apiospermum* has been rarely reported in the literature. In 2004, Thiagalingam et al. published the first case of sphenoidal sinus mycetoma due to *P. boydii* leading to OAS in a 92-year-old immunocompetent patient [9]. In 2017, Ippei Kishimoto et al. reported only the second case of *S. apiospermum*-related OAS secondary to a fungal nasal septal abscess in a patient with uncontrolled diabetes. In their study, *S. apiospermum* was detected by the polymerase chain reaction and tissue cultures [10]. Despite treatment with antifungal drugs and surgical resection of the lesion, this patient suffered bilateral vision loss in common with the present case. Their reports and the present one reflect the susceptibility of immunocompromised and elderly patients to invasive fungal infections.

In the present case, the patient first presented with dental abscesses in the mandibular region. His condition worsened, with rapid progression to pansinusitis, OAS and finally intracranial extension less than two months after the initial presentation, despite aggressive medical treatment and surgical drainage. Tissue culture from the septal wall revealed *S. apiospermum*, and pus and a nasal swab yielded methicillin-resistant *S. aureus*. Odontogenic sinusitis cannot be ruled out especially in immunocompromised patients with chronic dental abscesses. Previous research reported that sources of dental infections, including abscesses, cellulitis, periodontitis, extractions, root-canal therapy, periodontitis, dental braces and osteomyelitis, were potential causes of cerebral abscess [11-14]. Research also noted that odontogenic sinusitis was often under-recognized and refractory to treatment due to the polymicrobial, anaerobe-predominant nature of the infection [15].

The patient’s poorly controlled diabetes mellitus and history of recent extraction of abscessed teeth raised the suspicion of an infective cause of his symptoms, despite receiving treatment with IV amoxicillin-clavulanate (a broad-spectrum antibiotic and a β-lactamase inhibitor) and IV metronidazole that is generally effective against infections caused by anaerobes. Due to the patient’s history and rapid progression of the disease, which could indicate a possible fungal infection, empirical antifungal therapy was commenced at the time of his initial presentation, and steroid treatment was not started.

In this setting, the sensitivity of pathogenic fungi to the therapeutic agents was not investigated. Once *S. apiospermum* was isolated, IV treatment with amphotericin B was commenced. However, due to poor tolerance of the medication, the patient was switched to oral voriconazole on two occasions. Voriconazole is a broad-spectrum antifungal agent in vitro, with activity against *S. apiospermum*, *Aspergillus* spp. and *Fusarium* spp. [16]. McGinnis et al. studied the in vitro activity of voriconazole as compared with that of amphotericin B, fluconazole and itraconazole against several opportunistic molds [17]. They concluded that the level of minimal inhibitory concentration of voriconazole was lowest for 23 isolates of *P. boydii* [17]. However, in the present case, the patient’s infection progressed, despite treatment with voriconazole.

Despite early detection, prompt medical and surgical treatments and the adoption of a multidisciplinary approach, the patient’s vision could not be salvaged, and the lesion extended intracranially. In a previous study, Guarro et al. reported an extremely poor outcome in patients with central nervous system infections attributed to *S. apiospermum*, with mortality occurring in 51 of 66 patients [4]. The surviving patients had all undergone surgery and/or received systemic antifungal therapy [4].

## Conclusions

In immunocompromised patients presenting with vision loss and ophthalmoplegia due to OAS, life-threatening invasive fungal infections should be considered until proven otherwise. Prompt aggressive treatment and a multidisciplinary approach are crucial in such cases.
